# Comparative analysis of the physicochemical, proximate, and antioxidant characteristics of stingless bee (*Meliponula beccarii*) honey from modern and wild beehives in Ethiopia

**DOI:** 10.1002/fsn3.3861

**Published:** 2023-11-22

**Authors:** Taye Negera, Asfaw Degu, Fitsum Tigu

**Affiliations:** ^1^ Department of Microbial, Cellular and Molecular Biology Addis Ababa University Addis Ababa Ethiopia; ^2^ Oromia Agricultural Research Institute, Holeta Bee Research Center Holeta Ethiopia; ^3^ Department of Plant Biology and Biodiversity Management Addis Ababa University Addis Ababa Ethiopia

**Keywords:** antioxidant, beehive, honey, *Meliponula beccarii*, physicochemical, proximate, stingless bee

## Abstract

There is a dearth of information on the comparative studies of the physicochemical, proximate, and antioxidant properties as well as quality standards of stingless bee honey (SBH) in Ethiopia. Hence, this study was designed to assess and compare the physicochemical, proximate, and antioxidant properties of SBH, specifically sourced from *Meliponula beccarii*, and produced under both wild and modern apiary conditions at two distinct geographical locations. A total of forty‐six honey samples were meticulously collected from domesticated stingless bee colonies and naturally occurring wild nests at Wolmera and Cheliya districts. Pollen analysis unveiled eleven distinct bee plant species distributed across six families, with *Asteraceae* being the most prevalent, primarily represented by *Guizotia scabra* and *Vernonia amygdalina*. Notably, the physicochemical, proximate, and antioxidant properties of SBH collected from modern pot hives exhibited significant variances (*p* < .05) when compared to SBH from wild nests. Principal component analysis (PCA) delineated the differentiation of SBH sources based on both geographical location and the type of beehive. One‐way ANOVA corroborated these distinctions, underscoring significantly higher levels (*p* < .05) of ash, electrical conductivity, free acidity, hydroxymethylfurfural, sucrose, total phenolic content, total flavonoid content, and radical scavenging activities of SBH from modern pot hives in Wolmera. Whereas, Cheliya modern pot hives recorded higher values in pH, hydroxymethylfurfural and maltose contents compared to the wild nest SBH. Further analysis through Pearson correlation highlighted a strong positive association between total phenolic content and total flavonoid content with the antioxidant capacity of SBH. These findings underscore the significance of integrating modern pot hives to enhance the quality of SBH within Ethiopia's beekeeping sector.

## Graphical abstract

The SBH contains the most favorable combinations of mono‐ and disaccharide sugars. The antioxidant capacity in SBH showed a strong correlation with the total phenolic and flavonoid content. Modern beehives improved physicochemical, proximate, and antioxidant characteristics of SBH.

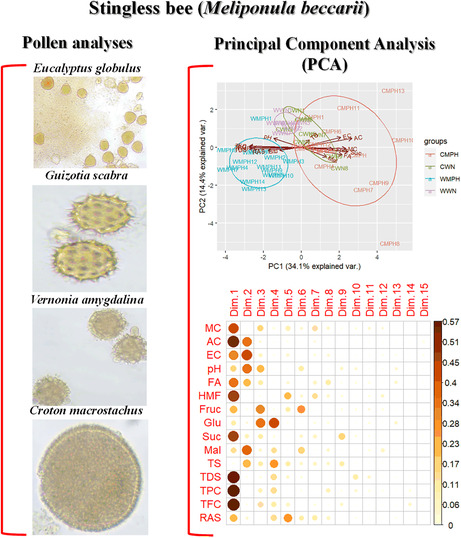

## 1 INTRODUCTION

Stingless bees (*Apidae: Meliponinae*) are a group of eusocial insects consisting of various known genera which are living in tropical and sub‐tropical regions of the world. Globally, over 56 genera and 600 species of stingless bees are known today and expected to be found in various ecological nests in the world, of which majority of them are living in feral colonies (Vit et al., 2004). Among many important species of stingless bees assumed to exist in the world, about six genera comprising twenty species are known to live in Africa (Eardley, 2005). Out of these, only five species are verified to be found in Ethiopia (Hora & Zewdu, 2013).

Honey is considered as a natural product composed primarily of sugars and water, enriched with various minor constituents like minerals, vitamins, amino acids, organic acids, flavonoids, phenolic compounds, and aromatic compounds (Santos‐Buelga & González‐Paramás, 2017). There are two primary origins of honey derived from different bee species worldwide: *Apis mellifera* honey and meliponines (stingless bees) honey. *Apis mellifera* honey stands as the most renowned and widely commercialized honey variety worldwide (Amano, 2002; Crane, 1991). In contrast, honey from stingless bees, although less prevalent, has a longstanding history of consumption within African communities, including Ethiopia. In Africa, it is predominantly utilized for its medicinal and traditional healing properties, often employed in the treatment of wounds, respiratory ailments, surface infections, diarrhea, and various other health conditions (Hau‐Yama et al., 2019).

Comparatively, SBH commands a higher demand and fetches better prices in the local market compared to Apis honey, making it a lucrative product in various regions of Ethiopia. Despite its premium pricing and recognized medicinal attributes, concerns persist regarding the quality and suitability of SBH for specific purposes, particularly in terms of physicochemical, proximate, and antioxidant properties. These aspects pose significant challenges for farmers, consumers, and the commercialization of the product, compounded by gaps in information regarding its production systems and composition (Gela et al., 2021). A comparative analysis of the physicochemical properties of the two honey types, stingless bee and *Apis mellifera*, revealed that honey from *M. beccarii* exhibited higher levels of free acidity and moisture content and a lower concentration of reducing sugar compared to honey from *A. mellifera* samples. Moreover, there was no statistically significant difference observed in the total phenol, flavonoid, and other antioxidant content between honey obtained from *M. beccarii* and *A. mellifera* (Tesfaye, 2018). However, verifying the authenticity of honey produced from *M. beccarii* is challenging given that the researchers utilized SBH available in the market.

In another investigation involving SBH samples collected from natural nests, a noticeable variance in the physicochemical parameters of SBH was observed, particularly in moisture content, total acidity, and hydroxymethylfurfural levels (Gela et al., 2021). These findings underscore the significant physicochemical disparities even within honey derived from the same *M. beccarii* species under natural nest conditions. However, there has been a dearth of comparative studies examining the physicochemical, proximate, and antioxidant properties of SBH from apiary conditions versus SBH obtained from wild nests. Notably, SBH is known to exhibit substantial concentrations of phenolic compounds, flavonoid content, and associated antioxidant capacity, highlighting its potential as a source of natural antioxidants (Biluca et al., 2017). Hence, this present study was conducted with the objective of assessing and comparing the physicochemical characteristics, proximate composition, and antioxidant properties of honey produced from stingless bees (*M. beccarii*) that were domesticated and managed under modern pot hives, as opposed to SBH obtained under natural, wild conditions.

## 2 MATERIALS AND METHODS

### 2.1 Description of the study area

The study was conducted at Holeta Bee Research Center's (HBRC) apiary sites namely Wolmera and Cheliya, West Shewa zone of Oromia, Ethiopia. Wolmera district is located in the central highlands of Oromia about 30 km away from the capital city of Ethiopia, Addis Ababa. It's found 9° 00’ N latitude and 38° 30′ E longitude, with an elevation of 2200–2600 meters above sea level.

Cheliya district is located 184 km from the capital city Ethiopia, Addis Ababa. The geographical location is between 8°48′0” N to 9°10′30” N latitude and 37°10′0″ E to 37°3′50″ E longitude with an altitudinal range of 1387–2870 meters above sea level. Both areas are characterized by bimodal rainfall distributions and favorable weather conditions, with great potential for the growth of diverse plant species with various flowering seasons in a year.

### 2.2 Honey sample collection

**FIGURE 1:**
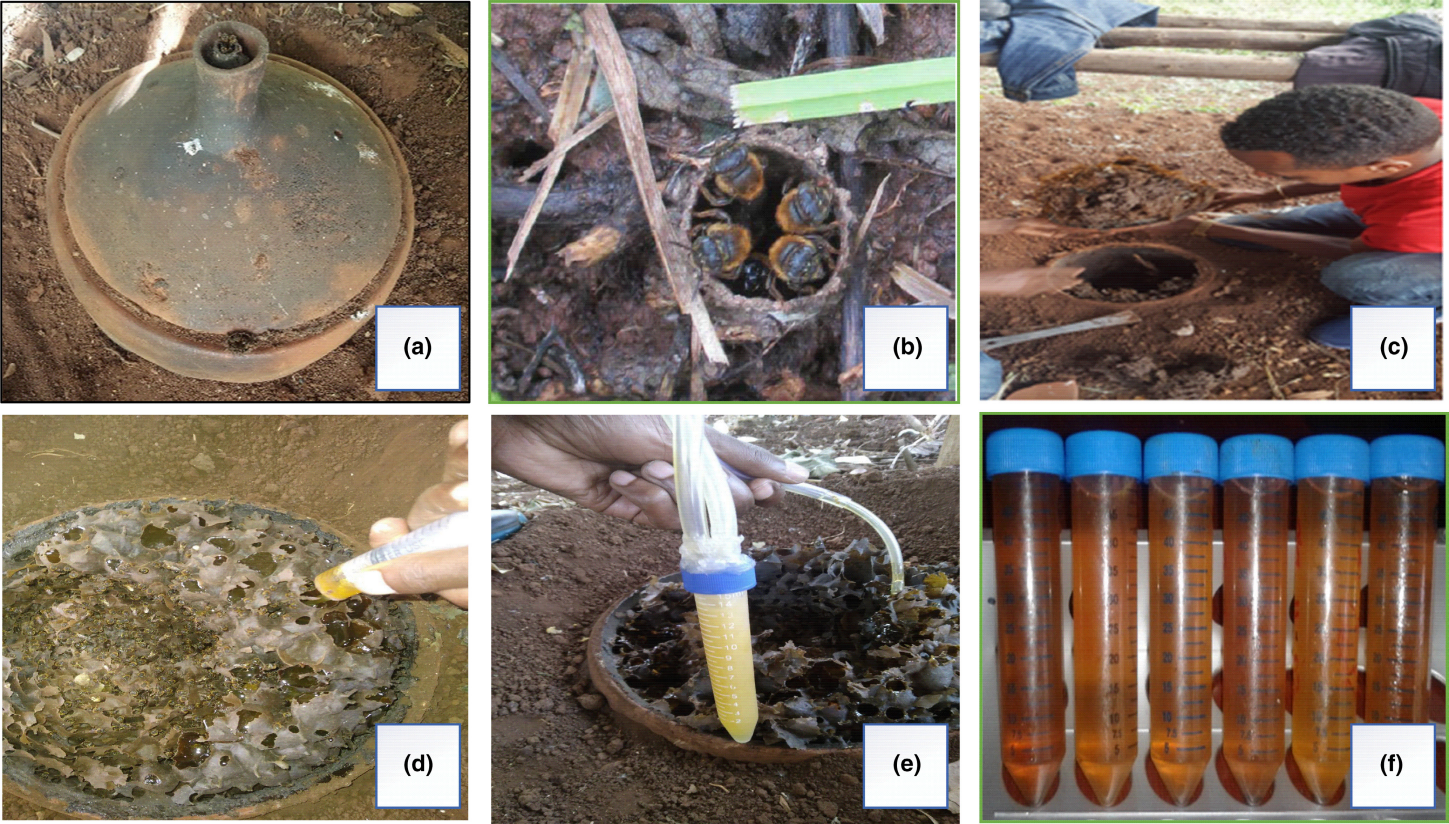
Stingless bee nesting and sample collection process. (a) Modern pot hive for stingless bees, (b) Ground‐nesting of stingless bees, (c) Process of opening pot hive, (d,e) Honey harvesting by pipette section pump, (f) Harvested pure honey.

For this experiment, previously domesticated and established stingless bee colonies (*M. beccarii*) were utilized as outlined in Gela et al. (2021). However, sample collection was executed using newly designed pot hives, illustrated in Figure 1a,b. Breeding of the stingless bee species involved employing queen‐rearing methods via colony splitting, a process carried out over three consecutive active seasons at HBRC. Thirty stingless bee colonies in modern pot hives (15 colonies from each site) were randomly selected and particular management practices were conducted in the two study sites prior to honey sample collection. A total of forty‐six samples, 15 samples each from modern pot hives of Wolmera and Cheliya and natural counterparts, 7 samples from Wolmera, and 9 samples from Cheliya wild nests were collected for comparison. The honey harvesting was conducted during two distinct honey flow seasons in December and May 2022. Harvesting involved extracting 50 mL of ripened, fresh, and pure honey directly from sealed honey pots using a pipette filler, as demonstrated in Figure 1c–e. These samples were then carefully transferred into airtight glass jars and stored in sterile falcon tubes, as illustrated in Figure 1f. The wild nest honey samples were carefully excavated from the nest chamber to obtain a combination of honey and pollen stores. Subsequently, all collected samples were meticulously packed in thermal boxes with ice and promptly transported to the HBRC laboratory. Immediate analysis of the samples was performed upon arrival.

### 2.3 Pollen analysis

The botanical origins of honey were identified using the method reported elsewhere (Von Der Ohe et al., 2004). About 10 g of honey sample was weighed and placed in a 50 mL centrifuge tube and dissolved by adding 20 mL of distilled water. After 10 min of certification at 12298 × *g*, the supernatant liquid was removed. The content was dissolved with 20 mL of water, and the supernatant was removed by centrifugation at 12298 × *g* for 10 min. The resulting solid content was carefully transferred to a sterile slide and sited on a hot plate for drying. Then, the dried sample was taken, a drop of glycerol jell was added and mounted on a microscope with a microscopic slide and cover slip. The botanical origin of the honey sample was examined under the microscope and the picture of the pollen was taken by the camera connected to the microscope (Carl ZEISS microscope). The pollen source of the plant was identified using reference slides and publications of pollen atlas (Adgaba, 2007).

### 2.4 Physicochemical and proximate analyses

The physicochemical characteristics and proximate composition of the honey were performed by standard protocol. Each set of experiments was performed in triplicates and the mean and standard error were reported.

#### 2.4.1 Moisture content (MC)

Moisture content was qualified by the AOAC method using Abbe refractometer (ABBE‐ 5 Bellingham Stanley Ltd) at 20°C temperature and the result in percentage (Helrich, 1990). The sample was homogenized, covered the surface of the prism evenly with the sample and refractive index reading was taken after 2 min using distilled water (1.3330) as a reference. The refractive index reading was converted into moisture content (g/100 g) using AOAC method.

#### 2.4.2 pH and free acidity (FA)

Ten grams of honey samples were dissolved in 75 mL of distilled water in a 250 mL beaker and stirred using magnetic stirrer. The pH was measured using digital pH meter (Model: NIG333, NAINA Solaris LTD). The free acidity of the honey sample was measured by the method reported elsewhere (Bogdanov, 2009).

#### 2.4.3 Electrical conductivity (EC)

Twenty gram dry matter of honey was dissolved in 100 mL of distilled water and the electrical conductivity was measured by using an electrical conductivity cell (BANTE Instrument‐520 conductive and temperature meter). First 0.745 g of KCl was dried at 130°C, dissolved in distilled water in a 100 mL flask and top up to 100 mL with distilled water. From the KCl solution, 40 mL was transferred to a beaker and connected to the conductivity cell. The cells were rinsed thoroughly with KCl solution and immerse the cell in the solution together with a thermometer. Electrical conductance reading was taken in millisiemens (mS) at equilibrium temperature (20°C) as described elsewhere (Bogdanov et al., 2002). The cell constant K was calculated using the following formula:

(1) $$K=\frac{11.691\times 1}{\;G}$$

Where:

K = the cell constant in cm^−1^.

G = the electrical conductance in mS, measured with the conductivity cell.

11.691 = the sum of the mean value of the electrical conductivity of freshly distilled water in mS.cm^−1^ and the electrical conductivity of a 0.1 mol/L potassium chloride solution, at 20°C.

#### 2.4.4 Hydroxymethylfurfural (HMF)

UV–Vis (6800) spectrophotometer (JENWAY) was used to measure the HMF as the method reported elsewhere (Bogdanov, 2009). Five gram sample was mixed in 25 mL of distilled water and absorbance at 284 and 336 nm against a filtered solution treated with NaHSO_3_ was recorded. Wavelength difference between the clear aqueous honey solution and the same solution with NaHSO_3_ was taken to avoid interference of other components. The HMF content was calculated after subtraction of the background absorbance at 336 nm. HMF per100 g of honey = (A284 − A336) × 14.97 × 5 g of the sample. The results expressed in milligram of the substance per kilogram of honey (mg/kg).

#### 2.4.5 Ash content (AC)

The ash content was measured by the standard protocol indicated in (Marchini et al., 2007). First, the crucibles were preheated at 300°C for 25 min and allowed to cool down and weighted to 0.001 g (M_1_). Ten grams of stingless bee honey sample was placed into a preheated and weighed crucible with two drops of olive oil and kept on a hot plate for gently heating until the sample was completely carbonized. The sample was burned in an electric muffle furnace at 600°C for 5 h until constant mass and the weight was taken as M_2_. Finally, the ash content (g/100 g) was calculated by the formula described below (Gela et al., 2021).

(2) $$\mathrm{Ash}\;\text{content}\;\left(\frac{g}{100g}\right)=\frac{M2-M1}{\;M0}\times 100$$

Where;

M_1_ = Mass of empty crucible.

M_2_ = Mass of the ash and crucible.

M_0_ = Mass of the sample taken for the test.

#### 2.4.6 Total dissolved solids (TDS) and total solids (TS)

The total dissolved solids (°Brix) was determined by using a refractometer (Q767‐B) at 20°C. The percentage of total solids content was calculated from the equation described below (Kamal et al., 2019).

(3) $$\mathrm{TS}\;\left(\%\right)=100–\text{Moisture content}$$

#### 2.4.7 Sugar contents analysis

The sugar contents of the honey sample including glucose, fructose, maltose, and sucrose were analyzed by HPLC system equipped with analytical stainless steel column composed of amine modified silica gel with 5–7 μm particle size (250 × 4.6 mm) and an RID detector (Agilent), using a mobile phase of acetonitrile: water ratio of 800 mL: 200 mL at 1.3 mL/min, 10 μL injection volume, and 30°C column temperature. The honey sugar contents were identified by comparison of their elution times with authentic reference standard sugars (99.5% purity, Sigma Aldrich) (Bogdanov, 2009).

### 2.5 Antioxidant properties

#### 2.5.1 Total phenolic content (TPC)

Folin–Ciocalteu method was used to determine the total phenolic content of honey samples (Meda et al., 2005). A 0.5 mL of honey solution (0.5 g/mL) mixed with 2.5 mL of Folin–Ciocalteu reagent for 5 min. In the mixture, 2 mL of sodium carbonate solution (75 g/L) was added and incubated at room temperature 30°C for 2 h. Further, 0.8 mL of 7.5% sodium carbonate was added in the mixture and agitated for 30 min in dark, and centrifuge at 1339 × *g* for 5 min. Both the honey sample and blank as a control were measured at 765 nm using a spectrophotometer. The total phenolic content in the honey sample was expressed as milligram of gallic acid equivalents (GAE) per 100 g weight of honey fitted with linear equation taken from the standard gallic acid calibration curve.

#### 2.5.2 Total flavonoid content (TFC)

Total flavonoid content was determined by the method described in Chua et al. (2013). A 5 mL of honey solution (0.5 g/mL) was mixed with 5 mL of 2% AlCl_3_ and incubated at 25°C for 10 min. UV–Visible spectrophotometer was used to measure the flavonoid‐aluminum complex formation at 415 nm wavelength. The total flavonoid content was determined based on a standard curve using quercetin, and the result was expressed as quercetin equivalent/100 g of honey.

#### 2.5.3 Free radical scavenging activity (RSA)

The percentage of free radical scavenging activity (RSA) of stingless bee honey was determined by the common chemical, 1, 1‐diphenyl‐2‐picrylhydrazyl (DPPH) (Chua et al., 2013). For assay preparation, 1.5 mL of DPPH solution (20 g/mL DPPH stock solution), was mixed with different concentrations (2.5–40 mg/mL) of 0.75 mL of methanol extracted honey samples. The mixtures were incubated at 25°C for 15 min together with ascorbic acid as a positive control and the absorbance was recorded at 517 nm. Each sample scavenge 50% of DPPH (EC_50_) was determined by the ascorbic acid calibration curve developed (0–10 mg/L) using the following formula below.

(4) $$\text{DPPH scavenging activity}\;\left(\%\right)=\frac{A\;\text{control}–A\;\text{sample}}{\;A\;\text{control}}\times 100$$

### 2.6 Statistical analyses

All data sets were analyzed using descriptive statistics and tested using one‐way ANOVA and Pearson correlation coefficient. Principal component analysis (PCA) was computed using the R‐software environment R 4.2.1. All analyses were performed in triplicates and data were presented as mean and standard error. Analysis of variance is used to compare the means of two groups of honey.

## 3 RESULTS AND DISCUSSION

### 3.1 Botanical origin

**FIGURE 2:**
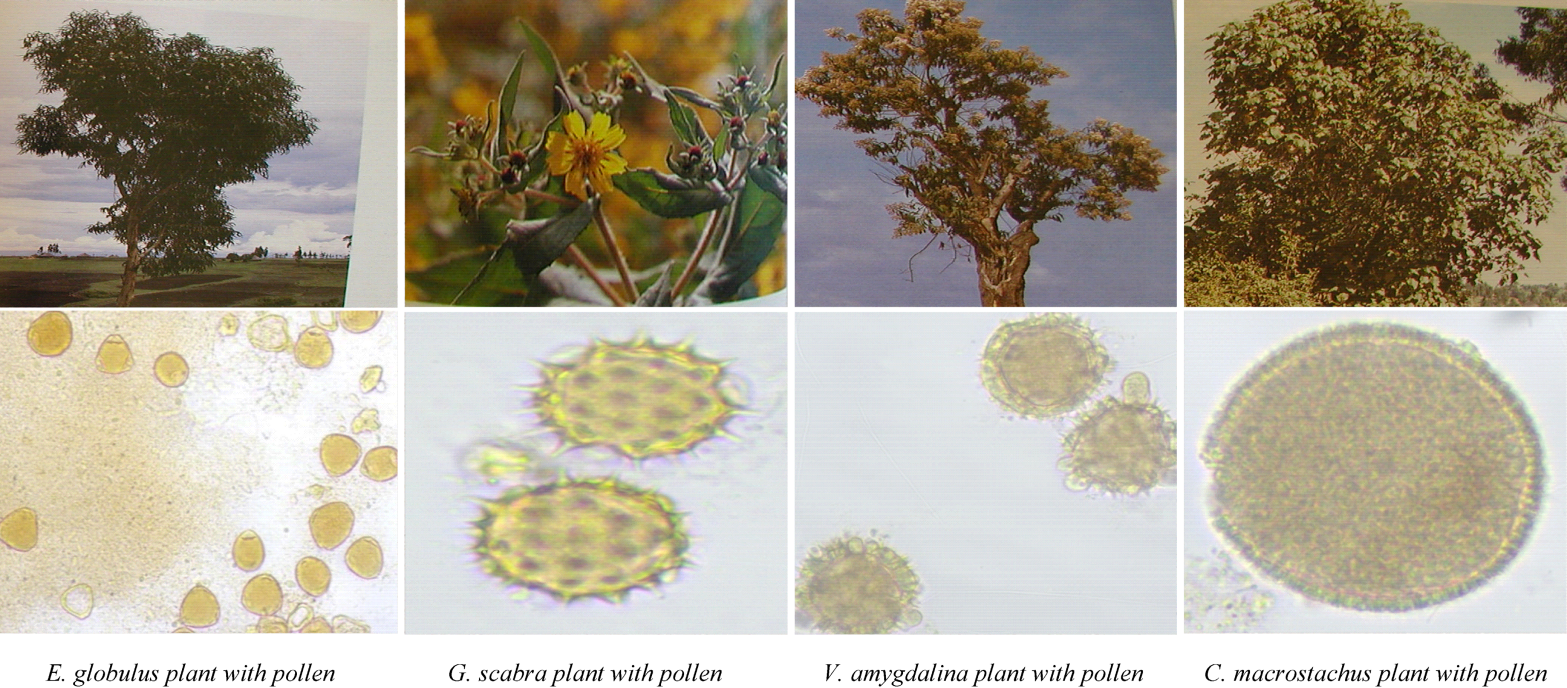
Stingless bee honey plant source, pollen shape, and size.

The pollen analysis indicates that fever trees *Myrtaceae* (*Eucalyptus globulus* and *Eucalyptus camaldulensis*) *Asteraceae* (*Guizotia scabra* and *Vernonia amygdalina*) and *Fabaceae* (*Trifolium burchellianum*), were predominant types of plants species recorded in honey samples collected from the study sites (Wolmera and Cheliya) of West Shewa zone of Oromia (Figure 2). While *Euphorbiaceae* (*Croton macrostachyus*) and *Vernonia amygdalina* (Cheliya sample), *Guizotia scabra* and *Eucalyptus camaldulensis* (Wolmera sample) were found to be secondary plant species as indicated in Table 1. The rest of the plants such as *Brassicaceae* (*Brassica carinata*), *Araliaceae* (*Schefflera abyssinica*)*, Croton macrostachys,* and *Trifolium spp* were identified as a minor plant source found in SBH samples.

**TABLE 1:** Stingless bee honey plant source, pollen shape and size.

District	Predominant (>45%)	Secondary (16–45%)	Important minor (3%–15%)	Minor (<3%)
Wolmera	*G. scabra, E. globulus*	*E. camaldulensis*, *V. amygdalina*, *Bidens spp*	*B. carinata*, *C. macrostachyus*	*Trifolium spp* unknown pollen
Cheliya	*E. globulus*, *E. camaldulensis*, *V. amygdalina*, *T. burchellianum*	*G. scabra*, *C. macrostachyus*	*S. abyssinica*, *Trifolium spp*	*T. vogelii*, *P. lanceolata*
Plant species	Pollen shape		Pollen grain size (μm)*	
*G. scabra*	Spheroidal, circular outline		19.5^d^	21.0^d^
*E. globulus*	Suboblate, triangular outline		18.5^d^	22.5^d^
*E. camaldulensi*	Spheroidal, triangular outline		17.5^e^	19.5^e^
*V. amygdalina*	Spheroidal, trilobed, subtriangular to circular outline		31.5^a^	33.5^a^
*Bidens spp*	Spheroidal, circular outline		19.0^d^	18.0^e^
*B. carinata*	Suboblate, circular outline		22.5^c^	27.0^c^
*Trifolium spp*	Spheroidal, circular outline		30.0^a^	30.5^b^
*T. burchellianum*	Spheroidal, round triangular outline		28.5^b^	29.0^b^
*S. abyssinica*	Spheroidal, subtriangular outline		20.5^c^	22.5^d^
*T. vogelii*	Spheroidal, circular to triangular outline		32.0^a^	26.0^c^
*P. lanceolata*	Spheroidal, circular outline		24.0	
*C. macrostachyus*	Spheroidal, circular outline		73.0	

*Note*: Means with different letters are not significantly different (*p* ≤ .05).

* Pollen grain size, first column is polar axis (P), second column is equatorial diameter (E).

According to our botanical origin of pollen analyses, diverse plant species were found in the SBH samples. Relatively, Cheliya honey samples had more plant species diversity than the honey collected from Wolmera district. These observations were in line with the previous study reported by Gela et al. (2021). Moreover, the pollen shape study revealed that most of the plants displayed a spheroidal pollen shape with circular outline. The polar axis and equatorial diameter of pollen grain size ranges from 17.5–32.0 and 18.0–73.0 (Table 1 and Figure 2).

The relative pollen frequency of honey samples indicated that *G. scabra* was a predominant pollen grain counted in honey samples collected from both districts of Wolmera and Cheliya. Additionally, *V. amygdalina* and *Eucalyptus* spp. were secondary pollen sources in both districts of West Shewa zone. These observations are similar to the finding of Gela et al. (2021). The substantial abundance of bee forages, particularly, *Guizotia and Trifolium species* might be due to the heavy rainy season (July to September). These plants bloom shortly after the end of the main rainy season in most highlands of Ethiopia as reported by Addi and Bareke (2019).

*Eucalyptus* spp. is the major bee forage that was found dominantly in honey samples collected from Wolmera and Cheliya districts of West Shewa Zone (Addi & Bareke, 2019). Bareke and Addi (2019) reported that *V. amygdalina* flowers offer ample pollen and nectar for stingless bees and honeybees from January to March, whereas *Bidens* spp. and *T. burchellianum* are prevalent during September to October.

Similarly, *Eucalyptus* spp. is a dominant plant species that commonly appeared from March to April at Wolmera district as reported by Bareke and Addi (2019). Vernonia honey is one of the monofloral honey that predominantly comprise *V. amygdalina* with a pollen frequency of 84.45% (Yadeta, 2019). Other bee forages that contributed to Vernonia honey were *Eucalyptus*, *V. auriclifera* and *Caesalpinade capetala*. Thus, the Vernonia honey tends to have unique color, aroma, taste and uniform and fine crystallization pattern (Bareke & Addi, 2019).

### 3.2 Physicochemical properties of SBH

**FIGURE 3:**
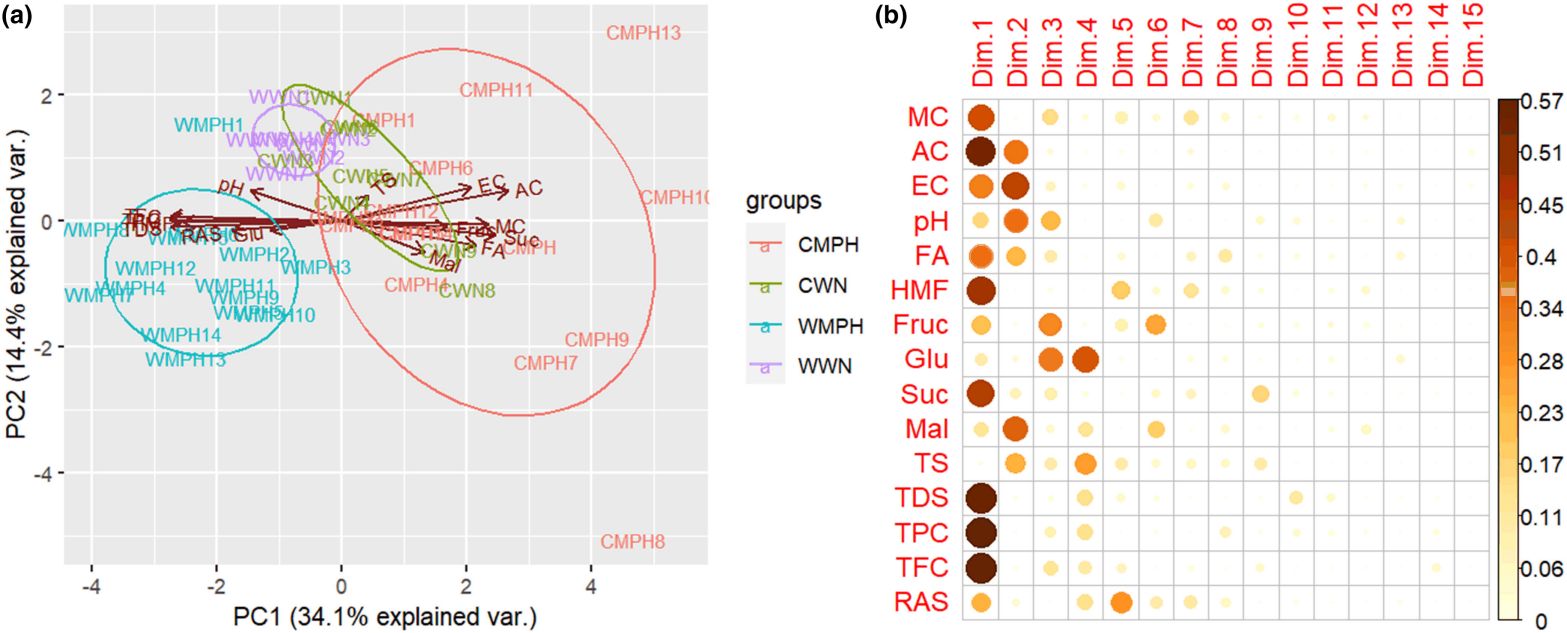
(a) PCA biplot based on all measured parameters for both modern pot hive and wild nest at Wolmera and Cheliya district. Percentage of the variance captured by each PC is given close to each respective axis. (b) List of contributing variables for each dimension, the size and color of the circle indicate the magnitude of contribution.

The physicochemical characteristics of the SBH extracted from the two districts of modern hive and wild nest were compared. In both districts of Wolmera and Cheliya, the mean values of FA and HMF showed statistically significant variation (*p* < .05) between the two kinds of honey samples (Table 2). This observation is further substantiated by the compelling evidence presented in the PCA plot (Figure 3a). The highest FA and HMF recorded from wild nest SBH of Cheliya district (87.20 ± 6.77) and modern hive of Wolmera district (7.30 ± 0.40), respectively. At Wolmera district, with the exception of MC and pH, all other physicochemical parameters showed statistically significant variations (*p* < .05) between the two kinds of SBH. The highest values of ash, EC, pH, FA and HMF recorded from SBH extracted from Wolmera modern hive with a values of 0.21 ± 0.01, 0.48 ± 0.01, 3.15 ± 0.03, 63.04 ± 1.37 and 7.30 ± 0.4, respectively. While in Cheliya, only the mean values of pH, FA and HMF showed significant variation between the two kinds of honey.

**TABLE 2:** Mean comparison of physicochemical properties of SBH samples collected from West Shewa zone of Oromia, Ethiopia.

District	Source	Parameters (mean ± SE)							
		MC (%)	AC (%)	EC (mScm^−1^)	pH	FA (meqkg^−1^)	HMF (mgkg^−1^)	TS (%)	TDS (°brix)
Wolmera	MPH	26.43 ± 0.23b	0.21 ± 0.01a	0.48 ± 0.01a	3.15 ± 0.03a	63.04 ± 1.37b	7.30 ± 0.40a	62.78 ± 0.33^d^	82.21 ± 0.24a
	WN	27.60 ± 0.27b	0.08 ± 0.01b	0.28 ± 0.04b	3.10 ± 0.04a	58.20 ± 2.70c	5.35 ± 0.17b	73.10 ± 0.23^a^	80.30 ± 0.27a
Cheliya	MPH	29.90 ± 0.22a	0.23 ± 0.01a	0.51 ± 0.01a	3.13 ± 0.04a	64.19 ± 2.80b	4.13 ± 0.43b	65.35 ± 0.20^c^	75.45 ± 0.23b
	WN	30.00 ± 0.23a	0.25 ± 0.01a	0.50 ± 0.03a	3.03 ± 0.05b	87.20 ± 6.77a	3.7 ± 0.31c	70.57 ± 0.15^b^	76.20 ± 0.17b
Mean	‐	28.48 ± 0.24	0.19 ± 0.01	0.44 ± 0.02	3.10 ± 0.04	68.16 ± 3.41	5.12 ± 0.33	67.96 ± 0.23	78.54 ± 0.23

*Note*: Treatments with the same letter are not significantly different along column.

Abbreviations: AC, Ash content; EC, Electric conductivity; FA, Free acidity; HMF, Hydroxyl methyl furfural; MC, Moisture content; MPH, Modern pot hive; pH, pH value; SE, Standard Error; TS, Total solids; TDS, Total soluble solid (°Brix 20°C refractometers 0–90 range); WN, Wild nest.

The moisture content (g/100 g) of both SBH types within the same district has no variations, however, in between the two districts, statistically significant variations were recorded (Table 2). Although the moisture content has no significant variations, one has to notice that the SBH from modern hives has lower moisture value than that of the wild nest SBH. The higher moisture content in both kinds of SBH of Cheliya district may be associated with higher humidity in this area. The reported average rainfall value of Cheliya district is 1524 mm annually and for Wolmera district is 1100 mm. Previous study report indicated that the value of moisture content in SBH from Ethiopia were recorded from 25–35 (Gela et al., 2021).

The overall mean moisture content of the examined SBH was 28.48, surpassing both the Ethiopian standard (Ethiopian Standard, 2005) and the international standard for *A. mellifera* honey, which allow a maximum moisture content of 20 g/100 g in honey (Codex Alimentarius Commission, 2001). This may be attributed to the higher water content found in the nectars and fruiting bodies of bee forage available in humid tropical environments (Esa et al., 2022). The moisture content is an important characteristic to determine honey quality and it has a direct impact on the stability and resistance to spoilage. Which in turn extend the shelf life of the honey by enhancing the specific weight, maturity, viscosity, crystallization, and taste of the honey.

The mean ash content (g/100 g) of SBH extracted from modern hives has a significant difference with that of SBH extracted from wild nests (Table 2). This shows that the modern management practice in stingless bees significantly improves the ash content of the SBH as compared to wild SBH. The present findings are in agreement with Abu Bakar et al. (2017) that reported ash content in SBH produced by *Heterotrigona itama* recorded a very low amount between 0.15 and 0.67.

The highest ash content, 0.90 was recorded in SBH samples obtained from coconut plant honey (Lim et al., 2019), whereas the lowest, 0.02 was reported from multi‐flora plant origins using stingless bee species of *Melipona subnitida* (de Almeida‐Muradian et al., 2013). Ash content of the honey is associated with bee diets, particularly the forages types, species and nutrient composition.

Looking at the mean pH values between the sources of SBH types and across locations, there is no significant differences except in Cheliya district (Table 2). The mean pH value obtained from *M. beccarii* honey samples contradicts recent studies that reported a pH range of 3.40–3.90 (Gela et al., 2021). Organic acid composition found in SBH keeps the pH at lower value and maintains the acidic environment against contamination. The lower pH value of SBH is an important parameter to inhibit both the presence and growth of microorganisms and can also affect the texture, stability, and shelf life of honey (Chirsanova et al., 2021). Furthermore, the acidity of SBH is vital for honey extraction, processing and storage conditions. Honey from stingless bees has low pH and low total solute content due to the presence of various acids (Mato et al., 2003). Honey pH is also influenced by several factors such as sources of nectar, type of soil and chemical compositions of the plant product, and bee species (Sousa et al., 2016).

The values of FA reported in this study from *M. beccarii* were comparable with Malaysian SBH produced by *Heterotrigona itama* and *Geniotrigona thoracica* (Ng et al., 2021). However, the highest variations of FA (meq/kg) between the SBH types and districts were reported in this study. These differences may be associated with the bees management practice, time of harvest, ripeness of honey, sources of flower, geographical origins, and weather conditions, which would favor the biochemical reactions able to release acidic compounds in SBH (Ahmed et al., 2014) and the existence of acetic and gluconic acids with other compounds (Seraglio et al., 2019). In this study, the free acid values were in conformity with the internationally established standard (Codex Alimentarius Commission, 2001).

The EC (mS/cm) of SBH extracted from the modern hive at Wolmera district (0.48 ± 0.01) was significantly different (*p* = .001) from that of the EC values of wild SBH sample (0.28 ± 0.04). Likewise, the EC of SBH extracted from modern hive at Cheliya district has better value than that of the EC values of wild SBH sample, even though the statistical differences were not observed. The improvements of the EC values under modern hive conditions clearly show the contribution of the bee management practice in the place. Since EC is associated with ash content and acidity due to availability of ions, acids, and proteins in the honey samples. Our results in ash content, and FA also conform these findings. Furthermore, the significant differences observed in EC values of the two districts (Wolmera and Cheliya) might be associated with variations in the geographical, botanical, and entomological origins of the SBH samples as reported elsewhere (Moo‐Huchin, González‐Aguilar, et al., 2015).

With regard to the concentration of HMF (mg/kg), SBH collected from the modern hives of Wolmera and Cheliya districts is significantly higher than that of the concentration of HMF collected from wild nest SBH samples. In this study, the HMF values observed in modern hives indicate the importance of stingless bee domestication and management practice to improve the physicochemical properties of SBH including HMF.

The obtained results of HMF are within the ranges of the national (Ethiopian Standard, 2005) and international (Codex Alimentarius Commission, 2001) quality standards that permit a maximum of 40. The HMF is an excellent indicator of the honey quality in terms of freshness, degree of deterioration, possible cause of microbial spoilage and the storage conditions of the honey samples (Mairaj et al., 2008; Moo‐Huchin, Aguilar, et al., 2015). Thus, maintaining HMF concentration under modern apiary conditions is quite important.

The results presented in Table 2 indicate a significant difference in percent total solids between the two kinds of SBH and fluctuated from 62.78% to 73.10% in Wolmera district and 65.35% to 70.57% in Cheliya district under modern and wild nests, respectively. The findings of total solids were in line with the reports of Moulya et al. (2023) and they found 73.27%–82.33%. According to Babarinde et al. (2011), about 85% of the formation of total solids in honey were derived from the two important sugars, glucose and fructose.

The total dissolved solids of SBH extracted from the modern hive and wild nest have no significant differences, however, significant differences (*p* < .05) were observed among the two different locations. The °Brix of total dissolved solids obtained in this study ranged from 76.20 to 82.21, which is quite in line with the previous reports of Moulya et al. (2023) and Ya'akob et al. (2019), they reported 69.17 to 76.17 and 69.0 to 81.8, respectively. The total dissolved solids tells us the amount of sugars found in honey, the SBH extracted from the wild nest has more sugars content than the modern pots SBH. This might be due to the differences in bees' management practices and availability of bee forages.

### 3.3 Proximate composition of SBH

The comparative studies of the proximate compositions of the two kinds of SBH indicated that with exceptions of sucrose and maltose, other parameters such as fructose, and glucose have no statistically significant differences in both districts (Table 3). The fructose content of SBH under modern hives were recorded, 37.31 ± 0.38 and 37.30 ± 0.28% from Wolmera and Cheliya districts, respectively. Compared to the wild nest fructose content of SBH, except in Cheliya district, there are no statistically significant differences between the two sources of honey. The overall mean fructose content of SBH under both conditions are 37.58 ± 0.34 g/100 g which agrees with other findings in Brazil (de Almeida‐Muradian et al., 2013).

**TABLE 3:** Mean comparison of sugar profile of SBH samples collected from West Shewa zone of Oromia, Ethiopia.

District	Source	Parameters (mean ± SE)			
		Fructose (%)	Glucose (%)	Sucrose (%)	Maltose (%)
Wolmera	MPH	37.31 ± 0.38b	27.98 ± 0.27a	1.28 ± 0.21ab	0.62 ± 0.04c
	WN	36.80 ± 0.41b	28.10 ± 0.23a	0.92 ± 0.16c	0.94 ± 0.10bc
Cheliya	MPH	37.30 ± 0.28b	28.52 ± 0.38a	1.82 ± 0.24b	2.22 ± 0.35a
	WN	38.90 ± 0.30a	27.80 ± 0.38a	2.02 ± 0.22a	1.60 ± 0.40b
Mean	‐	37.58 ± 0.34	28.10 ± 0.32	1.51 ± 0.21	1.35 ± 0.22

*Note*: Treatments with the same letter are not significantly different along column.

Abbreviations: MPH, Modern pot hive; SE, Standard Error; WN, Wild nest.

Likewise, the concentration of glucose in the SBH samples collected from Wolmera and Cheliya districts under modern hive and wild nest has no significant differences (*p* > .05). However, the overall mean value of glucose content in SBH, 28.10 ± 0.32 g/100 g is in line with the previous study in Brazil (de Almeida‐Muradian et al., 2013). A notable aspect of this study is the higher abundance of fructose compared to glucose in the SBH. This abundance is indicative of high‐quality SBH, likely contributing to its syrupy texture and exceptional hygroscopicity, allowing the honey to maintain a liquid state for an extended duration.

At Wolmera district, the mean value of the sucrose (%) recorded from modern hive was significantly varied with the naturally extracted SBH with values of 1.28 ± 0.21 and 0.92 ± 0.16, respectively. Likewise, at Cheliya district, the mean value of the sucrose recorded from modern hive was significantly varied with the naturally extracted SBH with values of 1.82 ± 0.24 and 2.02 ± 0.22, respectively. The sucrose content in SBH samples adheres to both the national and international quality standards, which stipulate a maximum sucrose content of 10% and 5% for *A. mellifera* honey, respectively (Codex Alimentarius Commission, 2001; Ethiopian Standard, 2005). Although the quality standard for SBH was not legislated, the current findings was comparable with the proposed maximum (6%) concentration for the *M. beccarii* honey (Vit et al., 2004). The high sucrose content of SBH may be associated with right time collection of honey from apiary sites and natural nests for this study.

The maltose concentration of SBH extracted from modern hive and wild nest were compared. The study result revealed that the concentration of maltose from Wolmera and Cheliya districts were (0.62 ± 0.04 and 0.94 ± 0.10), and (2.22 ± 0.35 and 1.60 ± 0.4) in modern hives and wild nests, respectively. Statistically significant differences (*p* < .05) were observed between the two types of honey and across the districts. In our study, we observed the lowest maltose content in comparison to findings from a study on *Tetragonula laeviceps‐pagdeni* species honey, which reported an average maltose content of 37 ± 12 g/100 g and a range from 15 to 57 g/100 g g (Chuttong et al., 2016).

Generally, the current study reported a bit higher concentrations of fructose and glucose and almost similar concentrations of sucrose and maltose compared to the recent study conducted in Malaysia from *Heterotrigona itama* species of honey (Ooi et al., 2020). The sugar compositional differences in SBH depend on the flower utilization of the honey bees, ecological and climatic conditions are the primary reasons indicated elsewhere (Tornuk et al., 2013). However, this study was unable to examine trehalulose, a recently identified sugar in SBH as reported by Fletcher et al. (2020), due to the unavailability of appropriate laboratory resources. Therefore, we highly recommended it to the inclusion of this specific sugar in future research endeavors focused on SBH.

### 3.4 Antioxidant characteristics of SBH

Another important parameter used for the comparative studies of SBH samples collected from Wolmera and Cheliya districts is the antioxidant properties of honey produced from modern and wild beehives. In the Wolmera district, our study revealed that the total phenolic content (TPC), total flavonoid content (TFC), and radical scavenging activity (RSA) of stingless bee honey (SBH) from modern hives were recorded as 34.55 ± 0.60, 30.57 ± 1.89, and 67.26 ± 1.86, respectively. These values demonstrated significant differences when compared to the SBH from wild nests (Table 4). However, in Cheliya district, with the exception of the RSA, other antioxidant properties such as TPC and TFC do not have any significant differences between the two kinds of SBH. Since phytochemicals such as polyphenols are the main contributing factors for the antioxidants properties in honey, the particular bee management practices and the bee species had significant impact as indicated elsewhere (Shamsudin et al., 2022).

**TABLE 4:** Antioxidant properties of SBH samples collected from West Shewa zone of Oromia, Ethiopia.

District	Source of SBH	Parameters (mean ± SE)		
		TPC	TFC	RSA
Wolmera	MPH	34.55 ± 0.60a	30.57 ± 1.89a	67.26 ± 1.86ab
	WN	12.10 ± 0.92b	13.30 ± 0.86b	61.40 ± 5.30c
Cheliya	MPH	34.24 ± 0.76a	32.67 ± 0.84a	72.82 ± 1.44 b
	WN	32.30 ± 1.70a	34.50 ± 2.20a	77.80 ± 3.60a
Overall mean	‐	28.30 ± 1.02	27.76 ± 1.64	69.82 ± 2.3

*Note*: Treatments with the same letter are not significantly different along column.

Abbreviations: MPH, Modern pot hive; RSA, Radical scavenging activity (mg of Ascorbic acid equivalent /100 g of honey); SE, Standard error; TFC, Total flavonoid content (mg quercetin equivalent (QE)/100 g of honey); TPC, Total phenol content (mg of Gallic acid equivalents (GAE)/100 g of honey); WN, Wild nest.

The total phenolic content of this study is in line with the recent findings in Malaysia (27.33 and 55.86) (Shamsudin et al., 2022) and higher than the Amazon SBH (17–66) (da Silva et al., 2013). Furthermore, the great variations of phenolic compounds in this study may be associated with harvest season, weather, and processing conditions. While variations in the total flavonoid content of honey samples from different areas signifies high diversity of floral and agro‐ecologies from where the stingless bees collect nectar.

Besides total phenolic content, the flavonoid content of SBH in the current study is in line with *Heterotrigona itama* honey from three botanical origins in Malaysia, 10.70–25.71 (Shamsudin et al., 2022). The highest RSA activity was recorded in both sources of honey samples at Cheliya district than Wolmera. According to the current results of RSA activity of SBH from two districts agro‐ecologies and kinds of honey is higher than SBH from Malaysia (11.27–24.09) (Shamsudin et al., 2022).

### 3.5 Principal component analysis (PCA) and correlation analysis

In the context of Principal Component Analysis (PCA), the initial two principal components (PC1 & PC2), as visually presented in Figure 3a, were employed to delineate samples of SBH source (modern pot hive vs natural nest) based on their respective geographical origins (Cheliya and Wolmera district). Collectively, these two principal components accounted for a large portion of the overall variance, elucidating 48.5% of the total variance. Principal Component 1 (PC1) was the primary contributor, explaining 34.1% of the variance, while Principal Component 2 (PC2) added a further 14.4%. The PCA biplot depicted in Figure 3a reveals the segregation of honey samples originating from the Wolmera modern pot hive versus those derived from the Cheliya modern pot hive along PC1, as depicted by the enclosed ellipses. Moreover, the honey from the Wolmera modern pot hive distinctly separated from both Wolmera natural nest and Cheliya natural nest along PC2. Nonetheless, a noticeable overlap is observed in the PCA biplot between Cheliya modern pot hive and Cheliya wild nest, signifying that the honey source (modern pot hive vs natural nest) does not lead to a clear differentiation within the Cheliya district. Further insights from the loading plot in Figure 3b highlight that variables such as moisture content (MC), ash content (AC), hydroxymethylfurfural (HMF), sucrose (Suc), total dissolved solids (TDS), total phenolic content (TPC), and total flavonoid content (TFC) predominantly contributed to group separation along PC1, whereas electrical conductivity (EC) and maltose (Mal) were the primary contributors for group separation along PC2.

Significantly positive and negative Pearson's correlations (r value above 0.6) were observed between the physicochemical, and antioxidant properties of SBH (Table 5). Ascorbic acid found to have significant positive correlation with total phenol content **(** *r* = .63, *p* < .05). In addition, significant positive correlation coefficients were observed between the total phenol content and total flavonoid content (*r* = .88, *p* < .05). This indicates that the ascorbic acid substances in honey (phenolics and flavonoids) influence the antioxidant activity.

**TABLE 5:** Pearson's correlation coefficients for quantitative determinations of SBH physicochemical, antioxidant parameters and major sugars profile.

	AA	TPC	TFC	MC	AC	EC	pH	FA	HMF	Fru	Glu	Suc	Mal
AA	1	0.63**	0.55**	−0.21	−0.18	−0.08	0.28	−0.32*	0.24	−0.14	0.35*	0.51**	−0.23
TPC		1	0.88**	−0.35*	−0.35*	−0.28	0.21	−0.26	0.22	−0.12	−0.29	−0.33*	−0.18
TFC			1	−0.34*	−0.29*	−0.19	0.27	−0.33*	0.24	−0.28	0.2	−0.36*	−0.19
MC				1	0.37*	−0.31*	−0.06	0.25	−0.26	0.39*	0.09	0.38*	0.26
AC					1	0.93**	−0.01	0.24	**−**0.49**	0.21	−0.34*	0.37*	−0.07
EC						1	0.07	0.16	−0.31*	0.07	−0.29	0.31*	−0.16
pH							1	−0.60**	0.1	−0.05	0.32*	**−**0.48**	−0.46**
FA								1	−0.43*	0.21	−0.1	0.53**	0.51**
HMF									1	−0.37*	0.09	−0.30*	−0.25
Fru										1	0.26	0.32*	−0.048
Glu											1	−0.30*	0.03
Suc												1	0.33*
Mal													1

*Note*: All values are at a 95% confidence interval. The values at the level of significance are reported as.

Abbreviations: AA, Ascorbic acid; EC, Electrical conductivity; FA, Free acidity; Fru, Fructose; Glu, Glucose; HMF, Hydroxyl methyl furfural; Mal, Maltose; MC, Moisture content; Suc, Sucrose; TFC, Total flavonoid content; TPC, Total phenol content.

* Significant at *p* ≤ .05.

** Significant at *p* ≤ .01.

The higher TPC and TFC and strong positive correlation with antioxidant capacity was found in this study. This result is in agreement with the SBH reported in Malaysia and Australia (Zawawi et al., 2022). Ash content was found to have a strong positive correlation with electrical conductivity (*r* = .93, *p* < .001), indicating that ash and EC values depend on the mineral content of the honey. The pH value was found to have a negative correlation with FA (*r* = −0.60, *p* < .005), indicating that lower pH levels and higher free acidity in SBH.

Overall, the SBH produced under modern pot hives had much better physicochemical, proximate, and antioxidant properties and quality compared to the SBH harvested from the wild nests. These results suggest the adoption of modern pot hives for better SBH quality and productivity of the beekeeping sector in Ethiopia. However, the exceptional presence of higher concentrations of FA, TS, fructose, sucrose and RSA in wild Cheliya honey may be associated with the presence of diverse honey bees forage in Cheliya district than Wolmera district.

## 4 CONCLUSION

This study compared the physicochemical, proximate, and antioxidant properties of SBH collected from modern pot hives and wild nests in the West Shewa Zone of the Oromia Region. The results indicated that SBH from modern hives exhibited superior physicochemical, proximate, and antioxidant characteristics compared to SBH from wild nests. This was particularly evident in the enhanced physical, chemical, and nutritional values of modern hive‐extracted SBH. The study also highlighted significant differences in antioxidant properties between the two types of SBH, underscoring the impact of modern beekeeping practices and domestication on these attributes, a trend supported by PCA plot. Pearson correlation analysis revealed a strong positive correlation between total phenolic and flavonoid content and antioxidant capacity. This research is the first of its kind in Ethiopia, emphasizing the potential benefits of domestication and modern beekeeping for stingless bees and suggesting future exploration into individual bioactive compound analysis and diverse antioxidant mechanisms.

## AUTHOR CONTRIBUTIONS

**Taye Negera:** Conceptualization (equal); data curation (equal); formal analysis (equal); investigation (equal); methodology (equal); writing – original draft (equal). **Asfaw Degu:** Formal analysis (equal); writing – review and editing (equal). **Fitsum Tigu:** Conceptualization (equal); formal analysis (equal); investigation (equal); methodology (equal); supervision (lead); writing – review and editing (lead).

## CONFLICT OF INTEREST STATEMENT

The authors declare that they have no conflict of interest.

## Data Availability

All relevant data are within the paper and are available upon request from the authors.
